# Antitubercular Drug Resistance in Four Healthcare Facilities in North India

**Published:** 2011-12

**Authors:** Anamika Gupta, Jitendra Prasad Mathuria, Surya Kumar Singh, Anil Kumar Gulati, Shampa Anupurba

**Affiliations:** ^1^Department of Microbiology, Institute of Medical Sciences, Banaras Hindu University, Varanasi, India; ^2^Department of Microbiology, Manipal College of Medical Sciences, Pokhara 977, Nepal; ^3^Department of Endocrinology and Metabolism, Institute of Medical Sciences, Banaras Hindu University, Varanasi, India

**Keywords:** Acquired immunodeficiency syndrome, Antitubercular drugs, Diabetes, Drug resistance, HIV, Tuberculosis, India

## Abstract

Tuberculosis (TB) is a major public-health problem in India, having the highest number of incident and multidrug-resistant (MDR) TB cases. The study was carried out to appraise the prevalence of first-line anti-TB drug resistance in *Mycobacterium tuberculosis* (MTB) and its patterns among different types of TB patients from different settings in a province of North India. Of 3,704 clinical specimens, 345 (9.3%) were culture-positive, and drug-susceptibility testing was carried out for 301 MTB strains. A high level of primary and acquired drug resistance of MTB was observed in the region studied, with weighted mean of 10.5% and 28.08%, 12.81% and 29.72%, 17.12% and 29.94%, 11.97% and 27.84%, and 10.74% and 23.54% for rifampicin, isoniazid, streptomycin, ethambutol-resistant and MDR cases respectively. Drug resistance was significantly higher in pulmonary (p=0.014) and acquired drug-resistant TB cases (p<0.001). Any drug resistance (p=0.002) and MDR TB were significantly (p=0.009) associated with HIV-seropositive cases. An urgent plan is needed to continuously monitor the transmission trends of drug-resistant strains, especially MDR-TB strains, in the region.

## INTRODUCTION

India has the highest number of the incident tuberculosis (TB) and multidrug-resistant (MDR) TB cases; yet the factors contributing to emergence, spread, and containment of TB are not well-estimated. TB is a major public-health problem, particularly in developing nations where the prevalence of infection is 40% (1). The incidence has been accelerated by the HIV epidemic, the appearance of new genotypes, multidrug-resistant and extensively drug-resistant(XDR) strains of *Mycobacterium tuberculosis* (MTB). Development of drug resistance in the population has increased the possibility that TB may once again become an incurable disease.

In 2008, there were 9.4 million new TB cases (including 3.6 million women and 1.4 million cases among people living with HIV) throughout the world. Approximately 1.8 million people died from TB in 2008, of whom 500,000 were HIV-infected people. HIV-positive people co-infected with TB are 20-40 times more likely to develop active TB than people without HIV infection living in the same country (2).

In 2008, most of the estimated number of TB cases occurred in Asia (55%) and Africa (30%). The five countries ranking first to fifth in terms of total numbers of incident cases were India (1.6-2.4 million), China (1-1.6 million), South Africa (0.38-0.57 million), Nigeria (0.37-0.55 million), and Indonesia (0.34-0.52 million). An estimated 35% of TB cases worldwide were found in India and China alone. There were an estimated 0.5 million cases of MDR-TB worldwide in 2007. The countries that ranked first to fifth in terms of total numbers of MDR-TB cases in 2007 were India (n=1,31,000), China (n=1,12,000), the Russian Federation (n=43,000), South Africa (n=16,000), and Bangladesh (n=15,000). To meet the targets set in the global plan, diagnosis and treatment of MDR-TB need to be rapidly scaled up, especially in the three countries that account for 57% of global cases: China, India, and the Russian Federation (3).

The incidence of TB is the greatest among those with impaired immunity, such as people with HIV infection and diabetes. HIV is a very important risk factor that enhances the progression of active TB in people with latent TB infection (4). The lifetime risk of TB in immunocompetent persons is 5-10% but, in HIV-positive individuals, there is a 5-15% annual risk of developing active TB disease (5). Diabetes mellitus (DM), a metabolic disorder, weakens the immune system. The incidence of TB, particularly pulmonary TB (PTB), is higher in diabetics compared to non-diabetics (6).

Resistance in cultures from patients for which treatment had been administered for <1 month or not at all is known as primary drug resistance (PDR) while that from patients with one or more previous TB treatment episodes (for at least one or more than one month), including those with treatment failures and relapse, is called acquired drug resistance (ADR) (7). MDR-TB is defined as resistance to the two main first-line anti-TB drugs—isoniazid (INH) and rifampicin (RIF)—with or without resistance to any other drugs. XDR-TB is a form of TB caused by bacteria that are resistant to most effective anti-TB drugs and defined as resistance to at least RIF and INH and to any member of the quinolone family and at least one of the following second-line anti-TB injectable aminoglycosides: kanamycin, capreomycin, or amikacin (8). Both primary drug resistance andacquired drug resistance contribute to MDR/XDR TB (9).

MDR-TB is an emerging problem in the world. Several outbreaks of MDR-TB have recently been reported. The prevalence of MDR-TB in India is 3.4% in primary (new) TB cases and 25% in acquired cases (10). In the second global report of the World Health Organization/International Union Against Tuberculosis and Lung Disease followed in 2000 in 58 countries, the median prevalence of resistance to at least one drug among new TB cases was 10.7% (range 2-36%), and that of MDR-TB was 1% (range 0-14%). In the previously-treated cases, the median prevalence of resistance to at least one drug was 23% (range 0-94%) and that of MDR-TB was 9% (range 0-48%) (11,12).

Surveillance data on primary and acquired drug resistance in MTB are important to design TB-control programmes. Escalating HIV infection and diabetes and negligence in TB control have caused an increase in the incidence of TB over the last decade in both developing and developed countries (13,14). Moreover, several other factors, such as homelessness, poverty, lack of infrastructure in public health, and inadequate access to health services have played an important role in worsening the situation.

In the present study, we aimed at determining the prevalence of first-line anti-TB drug resistance and its patterns in MTB isolated from different types of TB patients of North India.

## MATERIALS AND METHODS

### Study settings

The study was conducted at the Department of Microbiology, Institute of Medical Sciences, Banaras Hindu University (BHU). Sir Sundar Lal Hospital, a tertiary-care hospital of BHU, has a vast catchment area, this being the only tertiary-care hospital in north-eastern Uttar Pradesh (UP) providing medical coverage to a population of over 15 crore in eastern UP, western Bihar, and adjoining areas of Madhya Pradesh and Nepal. Our mycobacteriology laboratory is equipped to perform culture and drug-sensitivity testing (DST) for MTB. The sputum samples were collected from selected TB centres based on the maximum frequency of patients attending those centres. These were Department of TB and Respiratory Disease and antiretroviral therapy (ART) centres of Sir Sundar Lal Hospital of BHU, Shree Shiv Prasad Gupta District Hospital, Kabir Chaura (a secondary-care centre), Swami Vivekanand Smarak Rajkiya Chikitsalaya, Bhelupura (a primary healthcare unit), and Integrated Counselling and Testing Centre (ICTC) of the Department of Microbiology, Institute of Medical Sciences, BHU (a tertiary-care centre). The duration of the study was 25 months from January 2008 to January 2010. It included samples from both inpatients and outpatients.

### Study subjects

The study included TB patients with or without any other additional complication, such as HIV-seropositivity/diabetes (based on their previous and current medical records). Information was collected from the medical files and compliance charts on demographic characteristics of patients, radiological studies, and sputum mycobacteriologic studies.

### Collection and transportation of specimens

Specimens were collected in disposable widemouthed containers which were made of clear thin plastic, unbreakable and leak-proof material. These were placed in a box which could withstand leakage of contents, shocks, and other conditions incident to ordinary handling practices. Those boxes were immediately transported to the laboratory.

### Specimens/mycobacterial strains

Both pulmonary and extra-pulmonary specimens from 3,704 clinically-suspected TB patients, i.e. sputum, bronchoalveolar lavage (BAL), gastric fluid and cerebrospinal fluid (CSF), endometrial tissues, pus, fine-needle aspirate, urine, pleural fluid, lymphnode biopsy, ascitic fluid, pericardial fluid, knee fluid, and sinus discharge, were collected and used for preparing smear. The smears were subjected to acid fast stain by Ziehl-Neelsen method and examined by light microscopy at 100x oil-immersion objective. Sputum, BAL, pus and urine specimens were decontaminated by modified Petroff's method using 4% NaOH (15,16) while decontamination of other specimens was not needed as those were collected aseptically. All the specimens were concentrated by centrifugation at 3,200 × g for 20 minutes. The supernatant was discarded, and a part of the sediment was used for culture. Isolated cultures were characterized by certain biochemical tests, such as heat-stable catalase, niacin accumulation, and susceptibility to *p*-nitro benzoic acid (PNB) (17).

### Drug-sensitivity testing

Isolated MTB strains were subjected to indirect DST by proportion method (PM), the gold standard for DST of MTB. Conventional Lowenstein-Jensen (LJ) medium was prepared as described earlier (15). DST was carried out on LJ medium according to the standard procedures of the laboratory, with the recommended critical concentrations of 40 µg/mL for RIF, 0.2 µg/mL for INH, 2 µg/mL for ethambutol (EMB), and 4 µg/mL for streptomycin (STR) (15,18). In brief, bacterial suspension for DST was prepared in the concentration of 1 mg/mL suspension (S1 suspension). S1 was further diluted 10-fold to obtain S2-S4. S1-S4 bacterial concentrations were respectively inoculated into drug-free and drug-containing LJ slopes using a 3-mm internal diameter wire-loop and incubated at 37 °C. Growth was recorded at 28 days and at 42 days as follows: +++ for confluent growth, ++ for more than 100 colonies, and 1-100 actual numbers of colonies. Susceptibility or resistance was recorded when the proportion of bacteria in drug-containing medium to that of drug-free medium was <1 or ≥1 respectively.

### Laboratory quality control/quality assurance

H37Rv (ATCC 27294) and a known MDR strain were used as controls. The laboratory supervisor examined the DST results. DST of 69 MTB strains (23%), which showed contamination, was repeated.

### Analysis of data

The proportion of resistant isolates per setting was calculated. The weighted mean for each setting was calculated by multiplying the number of resistant cases in each setting and the weighted case (number of cases in each setting divided by the total number of cases) of the same setting. Other results were analyzed with the SPSS software (version 12.0.1) (SPSS Inc., Chicago, IL, USA). The features of two groups were compared using the Z-test and of three groups by chi-square (χ^2^) test for the assessment of statistical significance. A p value of <0.05 was considered significant.

### Ethical issues

The study was approved by the ethical committee of the institution.

## RESULTS

Pulmonary (85.56%) and extra-pulmonary (14.44%) specimens from 3,704 clinically-suspected TB patients were collected. Statistically, a sample-size of approximately 3,704 was required for investigation of the prevalence of TB and MDR-TB in our region.

Of the 3,704 specimens, 345 (9.3%) were culture-positive, of which 333 (96.52%) were tubercle, and 12 (3.48%) were non-tubercle bacilli. Of the 333 MTB isolates, DST of only 301 was performed. Due to contamination, we were unable to read the DST results of 32 culture-positive MTB isolates. Further, the sufficient extent of MTB growth was not available to repeat DST.

### Drug resistance

DST for all the four first-line anti-TB drugs, i.e. RIF, INH, STR, and EMB, was performed. There were no significant differences between the resistance rates of STR (46.84%), INH (42.83%), RIF (38.53%), EMB (39.20%), and MDR (34.55%) (Fig.).

Primary, acquired and total drug resistance levels for RIF, INH, STR, and EMB are shown in Table 1. Almost similar differences were found in resistance proportions for the individual drug among all four settings. On average, resistance proportions in new and acquired resistant cases were the highest for STR (17.12% and 29.94% respectively) and INH (12.81% and 29.72% respectively) whereas for EMB and RIF, these were 11.97% and 10.55%, and 27.84% and 28.08% respectively.

Some differences were observed in the percentage of MDR prevalence. Among the new cases, it was the highest (25%) in the cases from the first and third settings, followed by the fourth (15.0%) and second (14.28%) settings. In the acquired cases, it was the highest in the second setting (68.82%), followed by the fourth (66.66%) and third (50.0%) settings whereas it was the lowest in the first one (45.45%) (Table 1).

**Fig.**
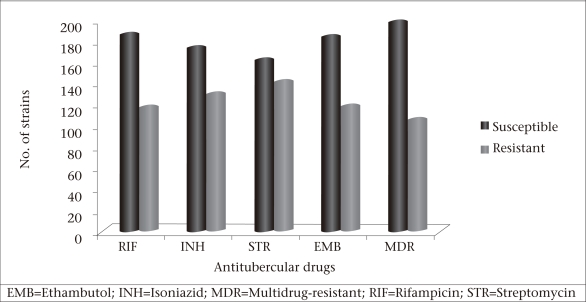
Results of drug-susceptibility testing for individual antitubercular drugs and MDR-TB cases

Of the 301 cases studied, 199 (66.11%) were male, and 102 (33.88%) were female. Of the 199 males, 104 (61.53%) showed resistance to one or more than one tested anti-TB drugs whereas 65 (38.46%) of the 102 females showed drug-resistant TB. The mean age at diagnosis of drug-resistant TB was 33 (range 4-75) years. Most cases of PTB were aged 21-40 years. Extra-pulmonary TB was documented mostly in the age-group of <10 years. Most common extra-pulmonary TB found was tubercular lymphadenitis (17.69%), followed by pleural TB (13.85%).

In the present study, resistance to any drug and multidrug resistance were analyzed against sex, age, nature of specimen, and type of resistance. Resistance was significantly higher in pulmonary (p=0.014) and ADR-TB cases (p<0.001). However, resistance was not significantly associated with any age-group or sex. In addition, MDR-TB was significantly higher only in the case of ADR-TB (p=0.0019) (Table 2).

Of the 301 cases for which DST was performed, 269 (89.37%) were both smear-and culture-positive whereas 32 (10.63%) cases were smear-negative but culture-positive. Of the 269 cases, which were both smear-and culture-positive, 151 (56.13%) were resistant to one or more tested anti-TB drugs. Of the 32 culture-positive cases, 18 (10.65%) were resistant to one or more tested anti-TB drugs. The prevalence of resistance to any drug and multidrugs was analyzed for the same categories and had no significant association (Table 3).

Of the 3,704 specimens, 82 (2.21%) were HIV-seropositive. Of the 82 HIV-seropositive specimens, 37 (45.12%) were culture-positive. Of the 37 culture-positive specimens, susceptibility testing was done for 32 specimens. By excluding the diabetic cases, the association of HIV and TB was analyzed in 294 (97.67%) cases. Of the 294 cases, 32 (10.88%) consecutive patients of TB were co-infected with HIV while 262 (89.12%) were only culture-positive for TB. Of the 32 HIV-TB cases, 26 (15.85%) were resistant to one or more tested anti-TB drugs, of which 10 (10.0%) were MDR. The prevalence of resistance to any drug and multidrug resistance was analyzed for the above two categories, and significant associations were observed (Table 3).

Of the 3,704 specimens, 26 (0.70%) cases were diabetics, along with smear-positivity. Of the 26 cases, only nine (34.62%) were culture-positive, and susceptibility testing was done for eight strains. By excluding the HIV cases, the association between diabetes and TB was analyzed only in 270 (89.70%) cases. Of the 270 cases, eight (2.96%) consecutive patients of TB had diabetes-related complication. Of the eight diabetic-TB cases, six were resistant to one or more anti-TB drugs, in which five were MDR. The prevalence of resistance to any drug and multidrug resistance was analyzed for the above two categories, and no significant association was found between them (Table 3).

**Table 1.** Observed drug resistance in new, previously-treated, and all TB cases from four different settings of Varanasi in North India

Setting	Resistant to														
	Rifampicin			Isoniazid			Streptomycin			Ethambutol			MDR-TB		
	PDR	ADR	All	PDR	ADR	All	PDR	ADR	All	PDR	ADR	All	PDR	ADR	All
	No. (%)	No. (%)	No. (%)	No. (%)	No. (%)	No. (%)	No. (%)	No. (%)	No. (%)	No. (%)	No. (%)	No. (%)	No. (%)	No. (%)	No. (%)
Department of Microbiology, IMS, BHU (3^0^ CC) (n=100*)	6(10.71)	30(68.18)	36(36.0)	10(17.85)	33(75.0)	43(43.0)	15(26.78)	31(70.45)	46(46.0)	8(14.28)	31(70.45)	39(39.0)	14(25.0)	20(45.45)	34 (34.0)
SS Hospital, IMS, BHU(3^0^ CC)(n=135*)	16(19.04)	37(72.54)	53 (39.25)	18(21.42)	38(74.50)	56(41.48)	24(28.57)	40(78.43)	64 (47.40)	18 (21.42)	36(70.58)	54(40.0)	12 (14.28)	35 (68.82)	47 (34.81)
TB Centre, Bhelupur(1^0^ CC)(n=32*)	7(35.0)	7(50.0)	14 (41.17)	6(30.0)	9(64.28)	15 (44.11)	6(30.0)	9(64.28)	15 (44.11)	4(20.0)	8(57.14)	12 (35.29)	5(25.0)	7(50.0)	12 (35.29)
TB Centre, Kabirchaura (2^0^ CC)(n=34*)	4(20.0)	9(75.0)	13 (40.62)	7(35.0)	8(66.66)	15(46.87)	7(35.0)	9(75.0)	16(50.0)	7(35.0)	6(50.0)	13 (40.62)	3(15.0)	8(66.66)	11 (34.37)
Weighted mean**	10.55	28.08	38.7	12.81	29.72	42.69	17.12	29.94	47.39	11.97	27.84	39.85	10.74	23.54	34.9

*No. of culture-positive specimens;

**Weighted mean (almost similar to an arithmetic mean) for all the four settings used in the study;

ADR=Acquired drug resistance;

BHU=Banaras Hindu University;

IMS=Institute of Medical Studies;

PDR=Primary drug resistance; 1^0^

CC=Primary Care Centre, 2^0^

CC=Secondary-care Centre; 3^0^

CC=Tertiary-care Centre;

Total no. of resistant strains=169;

Total no. of MDR strains=104

**Table 2.** Characteristics of tuberculosis patients for whom drug-susceptibility testing was done

Variable	No. (%)	Drug resistance	p value	MDR-TB	p value
		No. (%)		No. (%)	
Total cases	301	169		104	
Sex					
Male	199 (66.11)	104 (61.53)	0.0578	63 (60.57)	0.7452
Female	102 (33.88)	65 (38.46)		41 (39.42)	
Age (years)					
≤15	14 (4.65)	11 (6.51)	0.179	7 (6.73)	0.595
16-65	280 (93.02)	155 (91.71)		96 (92.30)	
>65	7 (2.32)	3 (1.77)		1 (0.96%)	
Nature of specimen					
Pulmonary	288 (95.68)	166 (98.22)	0.0140	103 (99.03)	0.311
Extra-pulmonary	13 (4.32)	3 (1.78)		1 (0.96)	
Type of resistance					
Primary resistance	180 (59.80)	71 (42.01)	<0.001	34 (32.69)	0.0019
Acquired resistance	121 (40.19)	98 (57.98)		70 (67.30)	

DST=Drug-susceptibility testing;

MDR=Multidrug-resistant

## DISCUSSION

DST is performed for several purposes, such as in relapse or retreatment cases, to change the drug regimen when resistance is suspected, or for undertaking drug resistance surveillance studies in a region/country.

Considering the resistance in all the four settings, on average, it was the highest for STR in new (17.12%) and in previously-treated cases (29.94%) while it was the lowest for RIF (10.55%) in new cases and for EMB (27.84%) in the previously-treated cases (Table 1). The same results were observed in a study in Russia, in which the highest resistance was observed for STR in new and previously-treated cases with 40.4% and 66.7% respectively whereas resistance to RIF and EMB was the lowest in new cases (13.5%) and in the previously-treated cases (60.0%) respectively (19). These observations clearly demon-strate the significance of critical monitoring of drug resistance pattern in a set-up, particularly where there is a high prevalence of drug resistance.

According to the World Health Organization (WHO), India is number one in terms of the prevalence of TB, and 3.4 million (17%) TB patients have developed multidrug resistance. Drug resistance surveys in Gujarat and Maharashtra (2005-2006) showed the prevalence of MDR-TB to be almost 3% among primary and 12-18% in previously-treated cases. It is estimated that the prevalence of MDR-TB may be three times greater than its incidence (20). MDR-TB in retreatment patients varies from 30% to 80% in different regions (21). In this study, on average, the resistance proportion of MDR-TB was high, with 34.61% among all the cases, 19.82% showing primary resistance, and 57.73% acquired resistance. We report a high rate of multidrug resistance in both newly-infected and previously-treated cases, which is in good agreement with earlier observations reported from India and some former Soviet Union countries (22-24). Among the new cases, the percentage of MDR prevalence was the highest (25%) in the cases from the first and third settings. In the case of the first setting, it could be because all the extra-pulmonary TB cases were from the same setting. In the third setting, being a primary healthcare centre, the prevalence of MDR-TB was the highest in new cases. In the acquired cases, it was the highest in the second setting (68.82%), followed by the fourth setting (66.66%). The second setting is a tertiary referral hospital, and the fourth setting is a secondary healthcare centre (district TB centre, Varanasi, India) where patients with more serious conditions may have presented, resulting in the highest number of acquired resistant cases. This study noted that the prevalence of MDR-TB was not significantly associated with age, sex, pulmonary TB, or extra-pulmonary TB.

Furthermore, resistance to any drug was significantly higher in the previously-treated patients (Table 2). The relationship between history of receiving anti-TB treatment and drug resistance has been clearly described in several studies (12,25,26). A significant difference in drug resistance between the new and the retreatment patients confirms the inefficiency of TB-control programmes. The use of irregular/improper anti-TB drugs during recent years has led to accumulation and multiplication of resistant strains. Notably, resistance to RIF, which did not show an increase in the new cases, was significantly elevated in the retreated cases. This reveals the fact that, with irregular treatment and in the presence of INH resistance, virtual monotherapy results in resistance to other agents as well (27).

**Table 3.** Drug resistance patterns among different types of tuberculosis patients

Type of disease	DST	Drug resistance	MDR-TB	Type of disease	DST	Drug resistance	p value	MDR-TB	p value
	No. (%)	No. (%)	No. (%)	(no.)	No. (%)	No. (%)		No. (%)	
				S+ C+ (269)	269 (89.37)	151 (89.34)	0.99	92 (88.46)	0.636
				S- C+ (32)	32 (10.63)	18 (10.65)		12 (11.53)	
HIV+ S+ C+	15 (4.98)	14 (8.28)	5 (4.81)	Total (301)	301	169		104	
HIV+ C+	3 (0.99)	2 (1.18)	1 (0.96)	HIV- C+ (32)	32 (10.88)	26 (15.85)	0.002	10 (10.00)	0.009
HIV+X-ray+ C+	13 (4.32)	9 (5.32)	3 (2.88)	HIV- C+ (262)	262 (89.12)	138 (84.15)		90 (90.00)	
HIV+ diabetes+	1 (0.33)	1 (0.59)	1 (0.96)	(excluded diabetic cases)					
X-ray+ S+ C+				Total (294)	294	164		100	
Diabetes+ S+ C+	7 (2.32)	5 (2.95)	4 (3.85)						
X-ray+ S+ C+	16 (5.32)	11 (6.51)	10 (9.6)	Diabetic+ C+ (8)	8 (2.96)	6 (4.17)		5 (5.26)	
X-ray+ S- C+	16 (5.32)	7 (4.14)	8 (7.69)	Non-diabetic+						
S+C+	230 (76.41)	120 (71.01)	72 (69.23)	C+ (262) (excluded HIV cases)	262 (97.04)	138 (95.83)	0.208	90 (94.74)	0.961
				Total (270)	270	144		95	

S+=Smear-positive;

C+=Culture-positive;

DST=Drug-susceptibility testing, MDR=Multidrug-resistant;

S-=Smear-negative

The proportions of drug resistance among the new and the previously-treated TB cases are important indicators for epidemiology of TB. The level of initial drug resistance is said to be an epidemiological marker to assess the success of the National TB Programme. This also influences the design of regimens to be employed and policy decisions. In a well-functioning TB-control programme, a low level of mistake in treatment can increase the probability of high resistance level among acquired resistant cases because drug resistance is a strong risk factor for recurrent TB. However, if a good TB-control programme is in place, proportion of the previously-treated patients among all TB patients should be low.

Overall, we found that the drug resistance to STR and INH in the new and the previously-treated cases was more frequent compared to other agents. Similarly, the first, second and third rounds of the WHO Global Projects and similar studies in Iran (eight years of surveillance) and Thailand have shown that the resistance to the above-mentioned agents was more common compared to the resistance to other first-line drugs (1,12,25,28). In general, resis­tance to STR and INH has been reported to be higher than EMB and RIF all over the world (29). However, other patterns of anti-TB resistance also exist. In a 15-year surveillance in Saudi Arabia, resistance to INH and EMB was more frequent than to other first-line drugs (30) whereas, in a study in Dhaka, Bangladesh, resistance to INH and RIF was found to be more frequent (31). Moreover, a prospective study set in the National Masan Tuberculosis Hospital in Masan, Republic of Korea, reported the enrollment and treatment of 19 patients with well-localized, cavitary pulmonary MDR-TB or XDR-TB with anti-TB therapy consisting of INH, RIF, EMB, pyrazinamide (Z), and STR. All recovered isolates of MTB were resistant to INH and RIF. Resistance to the first-line agents—EMB, STR, and Z—was observed in 73.7%, 36.8%, and 26.3% of isolates respectively (32).

In the present study, the prevalence of HIV in TB patients was 10.72% (37/345). A study in sub-Saharan Africa has recorded the HIV-seroprevalence rates of 50-70% in patients with TB (33). However, India has reported the HIV-seropositivity rates of 0.4-20.1% (34). This highlights the importance of effective guidelines developed by the WHO to control the emergence of TB co-infection in HIV/AIDS. In our study, resistance to any drug was significantly higher in the HIV-infected patients compared to the non-HIV patients. The same trend was also observed in the case of MDR-TB. According to the literature, infections with HIV and alcohol-abuse are important risk factors for the development of TB and the appearance of drug resistance in MTB infection (35).

The incidence of TB is higher in diabetics compared to the general population. The risk of developing an active TB infection is 3-7 times greater in persons with diabetes than non-diabetics (36). Each year, India accounts for one-fifth of the newly-diagnosed TB cases worldwide, of which almost half have diabetes (37). The prevalence of diabetes worldwide is close to 10%, and the relative risk of TB varies from 3% to >8% depending on the study (38). Furthermore, Stevenson *et al.* concluded that, in India, diabetes makes a substantial contribution to the burden of incident TB (39), and the association is particularly strong for the infectious form of TB. However, in our study, there was no significant relationship between TB and diabetes, which might be due to the enrollment of a low number of cases. The overall importance of diabetes as a risk factor for TB is still largely unidentified, although a recent study in Mexico concluded that, in the population studied, 25% of pulmonary TB was attributable to diabetes (36).

Our centre is an important referral centre in North India. However, our data may not necessarily be representative of the national population, especially with regard to new cases because only patients with more serious conditions may have presented to our centre. However, its findings will be indicative of the local population-related information for the previously-treated patients. A nationwide survey of drug resistance is required to achieve a more accurate assessment, management, and control of this deadly infectious disease.

A strong and cost-effective TB-control programme can reduce the incidence of drug resistance in the community. Some modifications should be made in running of the TB-control programme. For example, routine quality-assured DST for those patients who are at a high risk of resistance, especially failure cases, and those treated with second-line drugs should be done. In addition, we felt that the screening of all HIV/diabetic patients for TB and all TB patients for HIV and diabetes will help detect co-infected patients who require treatment for both the infections. This can be done by a good coordination and communication between TB and AIDS/diabetes-control programmes. These have potentially serious implications for TB control, and it must become a priority to use the existing knowledge about the association of TB with HIV/diabetes patients to initiate focused and coordinated actions, including new research in parts of the world where diabetes/HIV is epidemic and TB is endemic to properly inform public health and clinical practice. Standard chemotherapy with individualized drug resistance therapy, guided by conventional DST, might not be sufficient to control drug-resistant TB in northern India. Therefore, there is an urgent requirement of a plan to expand appropriate diagnostic and treatment services for patients with drug-resistant TB, especially MDR-TB, with or without having any additional complications, such as HIV/diabetes, throughout India and the world.

## ACKNOWLEDGEMENTS

The authors thank Dr. Susan van den Hof, KNCV Tuberculosis Foundation, The Hague, The Netherlands and Dr. Guangxue HE, National Center for TB Control and Prevention, China Center for Disease Control and Prevention, Beijing, PR China, for their valuable guidance in statistics.
